# Glioma tumor microenvironment and immunotherapy: past, present, and future

**DOI:** 10.1186/s40364-025-00863-2

**Published:** 2025-11-21

**Authors:** Josip Cvitković, Wen-Lu Tan, Tao Jiang, Zheng Zhao

**Affiliations:** 1 Beijing Neurosurgical Institute, Capital Medical University, Beijing, 100070 China; 2 Beijing Tiantan Hospital, Capital Medical University, Beijing, 100070 China; 3 Chinese/Asian Glioma Genome Atlas (CGGA/AGGA), Beijing, 100070 China; 4 Chinse Neuro-Oncology Genome Atlas (CGGA-CNS), Beijing, 100070 China

**Keywords:** Glioma, Immunotherapy, Tumor microenvironment

## Abstract

Gliomas constitute a major category of primary brain malignancies, characterized by limited therapeutic options and generally poor prognoses. Despite the promising outcomes of immunotherapies, particularly immune checkpoint inhibitors (ICIs), in various cancers, their clinical efficacy in gliomas has remained modest. This limited efficacy is largely attributed to the brain’s immune-privileged status and the profoundly immunosuppressive nature of the glioma tumor microenvironment (TME). These challenges underscore the urgent need to improve understanding of the glioma TME and to develop innovative strategies that enhance the effectiveness of immunotherapies. This review provides a comprehensive overview of recent advances in glioma immunobiology and immunotherapy, with emphasis on ongoing clinical trials and emerging combinatorial strategies. Current efforts to combine ICIs with modalities such as radiotherapy and chemotherapy are highlighted, aiming to remodel the TME, improve antigen presentation, and stimulate more robust antitumor immune responses. The evolving landscape of glioma immunotherapy offers renewed hope for enhanced patient outcomes.

**Clinical trial registration** Not applicable.

## Introduction

Glioma is among the most common primary malignancies of the central nervous system (CNS), accounting for approximately 80% of all primary CNS tumors [1, 2]. According to the most recent World Health Organization classification, adult-type diffuse gliomas are classified into three main types: *IDH*-mutant and 1p/19q-codeleted oligodendrogliomas (grades 2 and 3), *IDH*-mutant astrocytomas (grades 2–4), and *IDH*-wildtype glioblastomas (grade 4 only) [3]. These tumors are characterized by pronounced genetic and phenotypic heterogeneity, posing significant challenges for accurate diagnosis and effective treatment [4, 5]. The current standard of care (SOC) comprises maximum safe surgical resection followed by adjuvant radiotherapy and temozolomide (TMZ) chemotherapy. However, these interventions often produce only partial and transient responses, with frequent tumor recurrence and poor long-term survival. This highlights the urgent need for novel therapeutic approaches. 

The immune system plays a pivotal role in tumor surveillance and control. The brain’s status as an immune-privileged organ presents unique obstacles to effective antitumor immunity. The blood–brain barrier (BBB) limits immune cell infiltration into the tumor microenvironment (TME). Gliomas develop multiple mechanisms to evade immune detection, including reduced antigen presentation and the establishment of a profoundly immunosuppressive TME [6–9]. Downregulation of tumor-associated antigens (TAAs) can occur through epigenetic modifications and altered antigen presentation. Gliomas actively shape their microenvironment by expressing immune checkpoint molecules, recruiting regulatory T cells (Tregs) and tumor-associated macrophages (TAMs), and secreting immunosuppressive cytokines. These factors collectively promote immune evasion and facilitate tumor progression [10].

Recent studies have elucidated the complexity of the glioma TME, highlighting the dynamic interactions among tumor cells, immune cells, and stromal components. These insights have prompted efforts to develop immunotherapeutic strategies aimed at reactivating antitumor immunity. Immune checkpoint inhibitors (ICIs), particularly those targeting PD-1/PD-L1 and CTLA-4, have transformed the treatment of several malignancies [11, 12]. However, their efficacy in gliomas remains limited, largely due to the unique features of the CNS and glioma-specific immunosuppression. Current clinical trials are evaluating combination regimens that integrate ICIs with radiotherapy, chemotherapy, and targeted therapies, aiming to remodel the TME, enhance antigen presentation, and promote more effective immune responses.

Despite these challenges, the growing understanding of glioma immunobiology continues to drive the development of more effective immunotherapeutic approaches. This review summarizes current insights into the glioma TME and highlights recent advances in immunotherapy, focusing on emerging combination strategies and ongoing clinical trials.

## Immunosuppressive TME of glioma

**Fig. 1:**
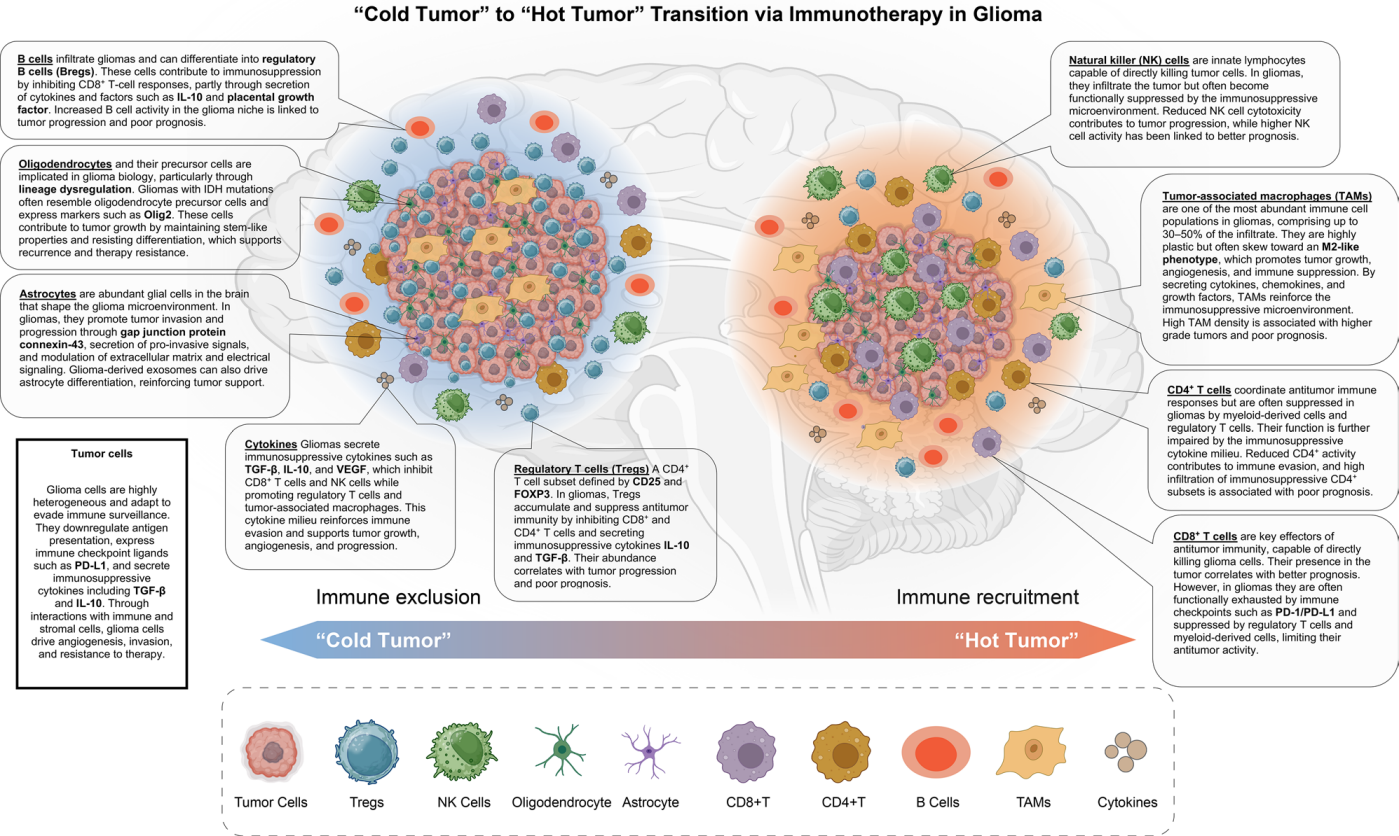
Immunotherapy-induced transition from ‘Cold’ to ‘Hot’ tumor microenvironment (TME) in Glioma. This schematic illustrates the immunological shift in gliomas from a “cold” TME, marked by immune exclusion, to a “hot” tumor state characterized by immune cell infiltration, an outcome targeted by immunotherapy. On the left, the cold tme contains a dense core of tumor cells surrounded by regulatory T cells (Tregs), natural killer (NK) cells, oligodendrocytes, and astrocytes, but shows limited infiltration by immune effector cells such as CD8+ T cells and B cells. This restricted immune presence contributes to tumor immune evasion and therapy resistance. On the right, following immunotherapy, the glioma transitions to a “hot” tumor, characterized by robust infiltration of CD8+ T cells, CD4+ T cells, B cells, tumor-associated macrophages (TAMs), and increased cytokine secretion. This immune activation promotes antitumor responses and supports immune-mediated tumor clearance. The cold-to-hot tumor transition reflects a central goal of modern glioma immunotherapy: converting immunologically “silent” tumors into “inflamed” ones capable of mounting effective immune responses

The TME of gliomas is highly complex and heterogeneous, comprising malignant glioma cells as well as a diverse array of stromal and immune cell populations, extracellular matrix components, and vasculature (Table 1) (See Fig. 1). This intricate ecosystem plays a pivotal role in promoting tumor progression and facilitating immune evasion. A defining feature of gliomas, particularly glioblastoma (GBM), is their capacity to establish a profoundly immunosuppressive TME that impairs the initiation and maintenance of effective antitumor immune responses.

**Table 1:** Immune cell populations in the glioma tumor microenvironment

Cell type	Function	Cell signature	Reference
Oligodendrocytes	The function of oligodendrocytes is to produce and maintain myelin in the central nervous system, which insulates nerve fibers and enhances the speed of electrical signal transmission.	MOG, MAG, ALCAM, CLDN11, CNDP1, DLL3, EDIL3	[5, 13, 14]
Astrocytes	The function of astrocytes is to support and maintain the blood-brain barrier, regulate blood flow, provide metabolic support to neurons, and participate in the repair and scarring of the brain and spinal cord after injury.	GFAP, ALDH1A1, ALDOC, AQP4, ATP1A2, CD44, CLU, GLAST, S100B	[5, 15, 16]
TAMs	The function of tumor-associated macrophages (TAMs) is to support tumor growth and progression by promoting angiogenesis, suppressing immune responses, and enhancing tumor cell invasion and metastasis through the release of cytokines and growth factors.	CD45, CCL2, CD11b, CD68, CX3CR1, IBA1, IL10	[17, 18]
Microglias	The function of microglia is to act as the primary immune cells of the central nervous system, involved in surveillance, phagocytosis of debris, and modulating inflammation in response to injury or disease.	CD45, P2RY12, ADORA3, ADRB2, BHLHE41, BIN1, CCL4, KLF2, NAV3, RHOB, SALL1	[18]
TAMos	Tumor-associated monocytes (TAMos) are key players in the glioma microenvironment, influencing tumor progression, immune responses, and treatment outcomes.	CD45, AREG, EREG, CD14	[19, 20]
Neutrophils	The function of neutrophils is to act as the first line of defense against infections by engulfing and killing pathogens, primarily through phagocytosis and the release of antimicrobial substances.	CD45, FCGR3B, CCR7, CEACAM8, CXCR2, FPR2, IL1R2, ITGAM	[17, 21, 22]
Dendritic cells	The function of dendritic cells is to capture, process, and present antigens to T cells, initiating and regulating the adaptive immune response.	CD45, CD11c, CD1C, HLA-DPA1, HLA-DPB1, HLA-DQB1, HLA-DRA, ITGAX, NRP1	[17, 23]
CD4+ T-cells	The function of CD4+ T cells is to coordinate the immune response by assisting other immune cells, such as B cells and CD8+ T cells, in recognizing and responding to pathogens or tumor cells.	CD45, CD3D, CD4	[24, 25]
CD8+ T-cells	The function of CD8+ T cells is to directly kill infected or tumor cells by recognizing specific antigens presented on their surface.	CD45, CD3D, CD8A, CD8B	[17]
NK cells	The function of NK (natural killer) cells is to recognize and eliminate virus-infected cells and tumor cells without the need for prior sensitization, through the release of cytotoxic molecules.	CD45, CD16, CD56, NKp46, NKp30	[23, 26, 27]
B cells	The function of B cells is to produce antibodies that recognize and neutralize pathogens, as well as to play a role in immune memory and antigen presentation.	CD45, CD79A, CD79B, BLK, CD19, CD20, FCER2, FCRLA, HLA-DPB1, HLA-DQA1, IGHG1, IGHG3	[21–23, 26]
Endothelial cells	The function of endothelial cells is to line blood vessels, regulating the exchange of gases, nutrients, and waste, while also playing a key role in maintaining vascular homeostasis and mediating inflammation and immune responses.	CAV1, CD31, CD34, CDH5, CLDN5, CLEC14A, ESM1, IFITM1, ITM2A	[21, 28, 29]

The glioma TME harbors various cell types, including resident glial cells (astrocytes and oligodendrocytes), neuronal cells, endothelial cells, and CNS-resident immune cells such as microglia. In addition, infiltrating immune populations, including TAMs, T cells, and myeloid-derived suppressor cells (MDSCs), are abundant within the TME [30]. These immune cells are frequently co-opted by glioma cells and reprogrammed toward immunosuppressive phenotypes that promote tumor growth, angiogenesis, and immune evasion [31]. For instance, TAMs in gliomas predominantly exhibit M2-like polarization, characterized by promotion of tissue remodeling, suppression of cytotoxic T-cell activity, and facilitation of tumor invasion and progression.

Additionally, the glioma TME is enriched with immunosuppressive cytokines and growth factors, including transforming growth factor-beta (TGF-β), interleukin-10 (IL-10), and vascular endothelial growth factor (VEGF). These soluble mediators impair the function of cytotoxic lymphocytes, including CD8+ T cells and natural killer (NK) cells, while enhancing recruitment and expansion of regulatory T cells (Tregs), thereby further reinforcing an immunosuppressive milieu.

The BBB, a critical structural component of the CNS, presents an additional barrier to effective immune surveillance by limiting the trafficking of immune cells into the brain parenchyma. Even when immune cells penetrate the BBB, their cytotoxic efficacy is frequently reduced by the immunosuppressive TME. Furthermore, glioma cells can directly engage immune checkpoints by expressing inhibitory ligands such as PD-L1, which bind to PD-1 on T cells, resulting in T-cell exhaustion and immune tolerance.

The interactions between glioma cells and immune components create a TME that is both immunosuppressive and spatially and functionally heterogeneous. Immune cells within this environment exhibit a spectrum of activation states, with some subpopulations promoting tumor progression while others attempt, but largely fail, to mount effective antitumor responses. This immune heterogeneity poses a significant challenge for immunotherapy, as gliomas exploit multiple redundant mechanisms to evade immune detection and destruction.

**Fig. 2:**
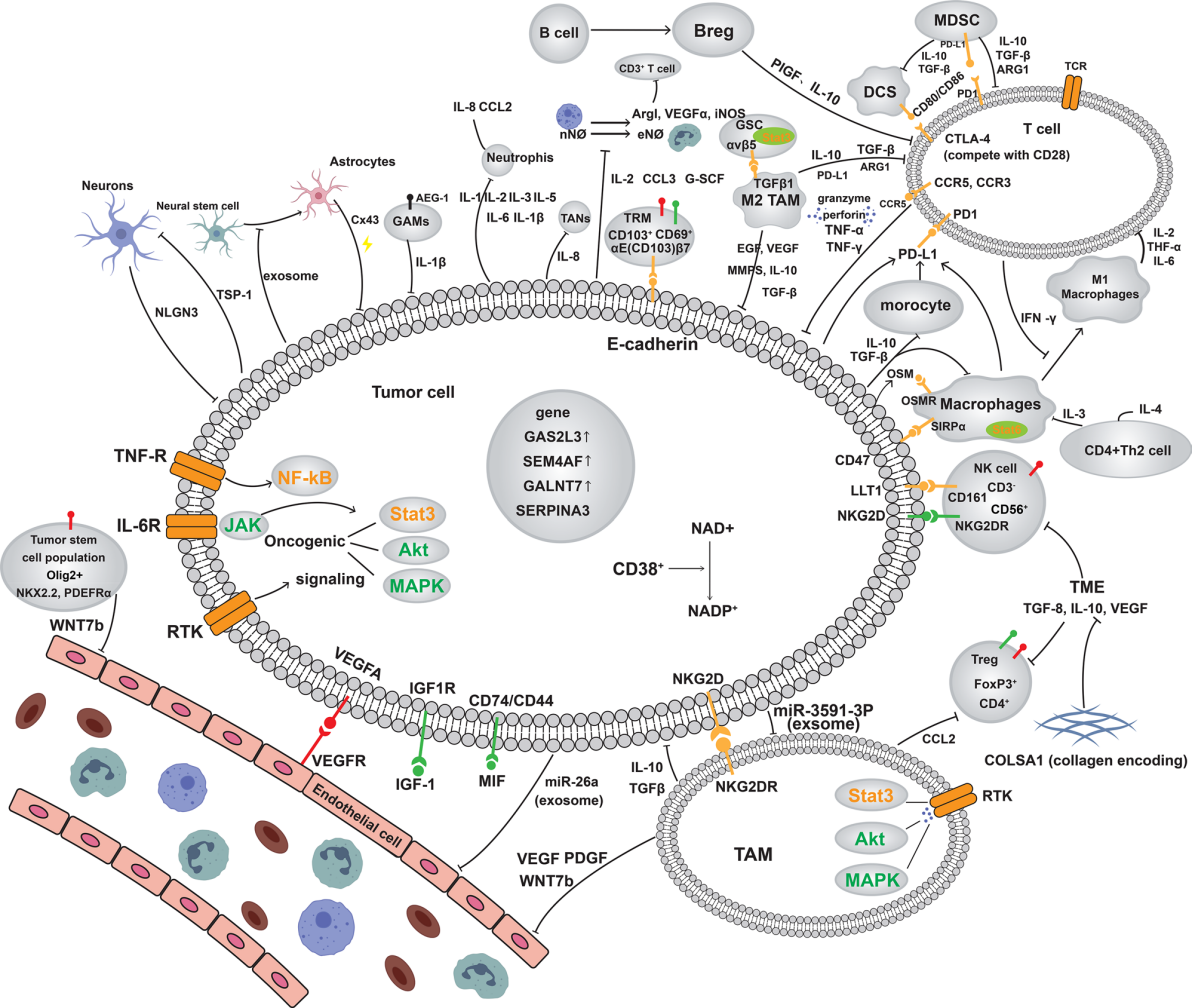
Immunoregulatory network in the glioma TME. This schematic illustrates the glioma TME driven by oncogenic signaling pathways such as JAK/STAT3, MAPK, and NF-κB, along with pro-angiogenic factors like VEGF/VEGFR. Tumor-associated macrophages, Tregs, Bregs, and myeloid-derived cells release immunosuppressive cytokines (TGF-β, IL-10, and ARG1), impairing the activity of cytotoxic T cells and NK cells. Interactions with neurons, astrocytes, and stem-like cells, including exosome release and paracrine signaling, further support tumor progression. These immune-modulatory mechanisms contribute to immune evasion, limiting effective antitumor immune responses and promoting sustained glioma growth

A comprehensive understanding of the cellular and molecular mechanisms underlying this immunosuppressive microenvironment is essential for designing effective strategies to restore immune function. Targeting these pathways may be crucial to unlocking the full therapeutic potential of immunotherapy in gliomas, ultimately improving patient outcomes (See Fig.2).

## Oligodendrocytes

Oligodendrocytes play a central role in glioma biology, particularly regarding lineage dysregulation during gliomagenesis. Recent studies have demonstrated that H3K27M-mutant gliomas predominantly consist of cells resembling oligodendrocyte precursor cells (OPC-like cells), highlighting the significance of the oligodendrocyte lineage in glioma pathogenesis [32]. These findings position oligodendrocytes as key contributors to the glial framework of malignant transformation, providing critical insights into the developmental aberrations driving tumorigenesis [33].

Single-cell transcriptome analyses have revealed that OPC-like cells are frequently in a proliferative state, highlighting their stem-like potential and contribution to tumor growth [34]. Notably, oligodendrocyte transcription factor 2 (Olig2) has emerged as a defining marker of glioma stem cells, which are strongly associated with tumor recurrence and resistance to conventional therapies [35]. Tumor progenitor populations in high-grade gliomas and oligodendrogliomas often express markers such as Olig2, Nkx2.2, and PDGFRα, supporting the concept that OPC-like cells act as critical drivers of glioma progression [36].

Furthermore, *IDH*-mutant gliomas recapitulate early stages of oligodendrocyte lineage development but exhibit a block in terminal differentiation. This differentiation arrest provides insights into the molecular mechanisms underlying glioma malignancy and identifies potential targets for therapeutic intervention [37]. Understanding and targeting dysregulated oligodendrocyte lineage pathways in gliomas, particularly in *IDH*-mutant subtypes, may provide novel strategies to overcome therapy resistance and tumor recurrence.

## Astrocytes

Astrocytes are integral components of the glioma microenvironment, significantly influencing tumor progression, cellular behavior, and intercellular communication. Aberrant regulation of microRNAs in astrocytes and microglia has been implicated in astrocytoma progression, suggesting that these cells modulate tumor dynamics [38]. A key molecular mediator of astrocyte-driven tumor promotion is connexin 43 (Cx43), a gap junction protein. Elevated C×43expression has been associated with the morphological transformation of glioma cells toward an epithelial-like phenotype, enhancing invasiveness [39]. Furthermore, Cx43-mediated intercellular communication between glioma cells and astrocytes is essential for the pro-invasive effects of C×43in vivo, promoting glioma spread and aggressiveness [40]. Astrocytes also modulate glioma cell migration by mediating electrical signaling within the extracellular matrix, particularly in three-dimensional collagen environments, further enhancing glioma cell motility [41]. Glioma-derived exosomes induce astrocyte differentiation from neural stem cells, demonstrating reciprocal interactions between glioma cells and the surrounding glial landscape [42]. Collectively, these findings highlight the multifaceted roles of astrocytes in shaping the glioma microenvironment. By promoting tumor invasion and influencing stem cell fate, astrocytes are central to glioma biology. Targeting astrocyte-tumor interactions may constitute a novel therapeutic strategy to inhibit tumor progression and improve clinical outcomes.

## Microglia

Microglia, the resident immune cells of the CNS, are critically involved in glioma progression and in modulating the TME. Microglia are among the most abundant immune cells in gliomas, constituting up to 70% of the tumor mass in high-grade gliomas, reflecting their prominent role in tumor biology [43–45].

Glioma cells secrete chemotactic and immunomodulatory factors that recruit and activate microglia and peripheral monocytes, establishing a substantial immune cell component within the tumor. These recruited cells correlate with enhanced tumor aggressiveness and poorer patient outcomes [46]. Upon activation, microglia promote tumor growth, invasion, and treatment resistance through the secretion of pro-inflammatory cytokines and growth factors, creating a supportive niche for tumor progression [47]. The interaction between glioma cells and glioma-associated microglia/macrophages (GAMs) is highly dynamic. GAMs exhibit diverse activation states, with the M2-like phenotype notably implicated in immunosuppression, tissue remodeling, and therapy resistance [48]. Activated microglia contribute to glioblastoma progression by promoting inflammation, angiogenesis, and cellular proliferation within the TME [49]. Emerging evidence indicates that targeting microglial function may constitute a viable therapeutic strategy. For instance, inhibition of microglial enhancer of zeste homolog 2 (EZH2) demonstrates antitumor effects in pediatric diffuse midline gliomas, highlighting microglia as a promising therapeutic target [50]. Pharmacologic agents, such as minocycline, that modulate microglial activation have also shown efficacy in reducing tumor grade and reshaping the TME in glioma models [51]. Collectively, these findings underscore the critical role of microglia in glioma pathophysiology and highlight the therapeutic potential of reprogramming microglial responses to enhance antitumor immunity and improve patient outcomes.

## Neural cells

Neural cells, including neurons and neural stem cells (NSCs)—play multifaceted roles in glioma biology, contributing to the TME as well as to tumor initiation, progression, and therapy resistance. While gliomas are believed to originate primarily from glial lineage cells, increasing evidence indicates that interactions with neural elements significantly modulate tumor behavior. Neurons within the glioma microenvironment engage in bidirectional communication with tumor cells, which can profoundly influence disease progression. Glioma cells release neurotransmitters and signaling molecules that alter neuronal excitability and activity, thereby creating a microenvironment conducive to tumor growth [52–54]. In turn, neurons support glioma proliferation and invasion by supplying trophic factors, synaptic inputs, and electrical signals that stimulate oncogenic pathways in tumor cells [55, 56]. This reciprocal interaction may also underlie glioma-associated epilepsy, a common and debilitating comorbidity observed in patients with glioma. NSCs constitute another key component of the glioma TME. Under normal physiological conditions, NSCs contribute to brain homeostasis and regeneration. However, in the context of glioma, NSCs may be reprogrammed or co-opted by tumor cells. Glioma cells activate and expand the NSC population, transforming these cells into tumor-supportive or even tumor-initiating cells [57].

Moreover, NSCs can promote glioma invasion through secretion of pro-invasive cytokines and remodeling of the extracellular matrix, thereby facilitating tumor dissemination and therapeutic resistance. Dynamic crosstalk between glioma cells and neural elements—both mature neurons and NSCs—creates a complex microenvironment that supports tumor aggressiveness. Deciphering the molecular and cellular mechanisms governing these interactions may offer novel therapeutic opportunities. Strategies that disrupt glioma–neural cell communication could impede tumor progression, reduce recurrence, and improve patient outcomes.

## Tumor-associated macrophages (TAMs)

TAMs are a crucial component of the glioma TME and play a central role in promoting tumor growth, progression, and metastasis [58–61]. TAMs are highly plastic and can adopt a spectrum of functional phenotypes, most notably the classically activated M1 and alternatively activated M2 states. M1 macrophages are generally pro-inflammatory and exert antitumor effects, whereas M2 macrophages are associated with immunosuppression, tissue remodeling, and tumor progression [62, 63]. The dynamic balance between these phenotypic states critically influences glioma progression and the tumor’s response to therapy [64]. In gliomas, especially GBM, TAMs constitute a predominant immune cell population within the TME, comprising approximately 30–50% of infiltrating immune cells and in some cases accounting for up to 70% of tumor mass [65, 66]. They contribute extensively to the immunosuppressive landscape through secretion of cytokines, chemokines, growth factors, and metabolic modulators that support tumor proliferation and survival [67]. TAMs are implicated in nearly every aspect of glioma biology, including tumor growth, invasion, angiogenesis, immune suppression, and metabolic reprogramming [68]. Notably, unlike many other solid tumors, the glioma microenvironment is particularly enriched with TAMs, highlighting their unique importance in glioma pathophysiology [69]. TAMs interact closely with glioma cells through various mechanisms, including the bidirectional exchange of exosomes. These exosomes modulate gene expression and immune signaling, further reinforcing the immunosuppressive TME and promoting glioma progression [70]. One key signaling axis involves SPP1 (osteopontin) and CD44, which facilitates glioma cell migration and invasion and is largely mediated by TAM-derived factors [71]. Moreover, TAMs support glioma stem cell maintenance and expansion, thereby sustaining the tumor’s self-renewal capacity and resistance to conventional treatments [72]. The clinical relevance of TAMs is underscored by their prognostic value in glioma. Increased TAM infiltration is frequently associated with higher tumor grade and poorer patient outcomes [73].

Given their dual role as mediators of tumor progression and modulators of the immune response, TAMs represent both a valuable biomarker and a promising therapeutic target in glioma. Therapeutic strategies that reprogram TAMs or inhibit their recruitment and pro-tumor functions hold significant potential to enhance treatment efficacy and improve patient survival.

## Tumor-associated monocytes (TAMos)

TAMos are key components of the glioma TME. Along with microglia and macrophages, TAMos represent a significant portion of the immune infiltrate in gliomas, shaping the tumor’s immune landscape and influencing both tumor progression and response to therapy [43]. The glioma microenvironment is primarily composed of tumor-associated microglia (TA-MG) and monocyte-derived macrophages (MDMs), with TAMos functioning as key players in this ecosystem [74]. TAMos in glioma exhibit remarkable plasticity, transitioning between functional states depending on tumor stage and microenvironment cues. In early-stage gliomas, TAMos adopt a pro-inflammatory “M1” phenotype, which is associated with antitumor responses. However, as disease progresses, TAMos shift toward an immunosuppressive “M2” phenotype, facilitating tumor growth, immune evasion, and therapy resistance [75]. This phenotypic transition is critical in determining how TAMs influence tumor dynamics during glioma progression.

Moreover, specific gene signatures identified in TAMos can help predict patient prognosis and guide treatment decisions, highlighting their potential as biomarkers in glioma [76]. TAMo infiltration of the glioma microenvironment has been linked to promotion of tumor growth and formation of an immunosuppressive niche, further exacerbating glioma progression and complicating therapeutic efforts [77]. Understanding the multifaceted roles of TAMos in glioma is essential for developing strategies aimed to modulate these cells, enhance therapeutic efficacy, and improve patient outcomes.

## Neutrophil

Neutrophils play a significant role in shaping the immune microenvironment of glioma. Neutrophils are recruited to glioma inflammatory regions by chemokines such as CXCL1, CXCL2, CXCL3, CXCL5, CXCL6, IL-8 (CXCL8), and IL-1β, which are secreted by glioma cells [78]. Tumor-associated neutrophils (TANs) are primarily attracted to tumor sites by IL-8 produced by glioma cells [79]. Within the glioma microenvironment, neutrophils promote cell proliferation, support glioma stem cells (GSCs), facilitate angiogenesis, and contribute to therapy resistance [80]. Notably, neutrophil infiltration correlates with high-grade gliomas, implicating their role in driving tumor progression and treatment resistance [81]. In particular, neutrophil accumulation has been linked to glioma progression and reduced efficacy of anti-VEGF therapies targeting tumor vasculature [82]. Neutrophils have been shown to enhance glioblastoma cell migration after biopsy, potentially complicating surgical resection and increasing metastatic potential [83]. Neutrophils also secrete elastase, an enzyme that promotes glioma cell proliferation, further reinforcing tumor aggressiveness [84]. High densities of infiltrating neutrophils are commonly observed in advanced-stage cancers, including gliomas, and are associated with poorer prognosis [85]. Together, these findings highlight the complex and multifaceted roles of neutrophils in glioma biology, underscoring their potential as biomarkers and therapeutic targets to improve treatment outcomes.

## Dendritic cells (DCs)

DCs play a crucial role in the immune microenvironment of gliomas, primarily through antigen presentation and activation of T cells. However, the glioma microenvironment suppresses DC function, hindering their ability to effectively activate T cells and initiate an antitumor immune response [86]. Despite this, DC presence in gliomas has been associated with expression levels of certain genes, such as COL5A1, which correlate with tumor progression and patient prognosis [87]. This association highlights the potential significance of DCs in glioma biology and their role in shaping the TME [88]. Although the role of DCs in glioma has only recently been recognized, their importance is increasingly acknowledged. DC-based vaccines, designed to enhance DC function and promote T-cell activation, have shown promise in modulating the glioma microenvironment and improving immune responses [89]. Conventional DCs (cDCs) are particularly important for initiating antitumor immunity, and their presence in the glioma microenvironment is essential for stimulating effector immune functions [90]. Different DC subsets have been identified in gliomas, further emphasizing their significance in tumor immunology [91]. Plasmacytoid DCs (pDCs), another DC subset, play a significant role in immune regulation within the glioma microenvironment. pDCs exhibit immunosuppressive functions in gliomas, and depletion of pDCs during early tumor progression significantly reduces regulatory T cells (Tregs) within the TME [92]. pDCs produce type I interferons, which are essential for modulating immune responses and maintaining immune tolerance [93]. These cells can be identified by specific markers such as CD123 and blood-derived dendritic cell antigen-2 (BDCA-2) [94]. In gliomas, pDCs infiltrate tumors alongside other immune cells, including CD8+ T cells, macrophages, T helper cells, Tregs, and immature DCs, highlighting their involvement in the complex immune landscape [95]. These findings underscore the multifaceted roles of DCs in gliomas, from antigen presentation to immune regulation, and their potential as therapeutic targets to improve glioma treatment.

## CD4+T cell

CD4+ T cells are integral components of the immune response within the glioma microenvironment. These cells play critical roles in orchestrating immune responses against tumors, yet their function is often compromised by the immunosuppressive microenvironment of gliomas. Studies demonstrate that glioma-associated myeloid cells can suppress CD4+ T-cell antitumor activity, thereby facilitating tumor progression and contributing to immune evasion [96].

Recent research has identified the gene DDOST as a key factor linked to the glioma microenvironment [97]. DDOST is negatively correlated with tumor-infiltrating CD4+ T cells and B cells, and positively correlated with the presence of cancer-associated fibroblasts (CAFs) and TAMos, further highlighting the complex immune landscape of gliomas. Additionally, SERPINA3, a protein highly expressed in gliomas, is associated with poor prognosis and immune suppression, highlighting the essential role of CD4+ T cells in resisting tumor progression [98].

The glioma microenvironment is characterized by predominant infiltration of both CD8+CD25- T cells and CD4+CD25+FOXP3+ Tregs. These Tregs are key drivers of immune suppression within tumors, dampening CD4+ T-cell antitumor responses and contributing to the immunosuppressive milieu that enables glioma survival and growth [99]. CD4+ T cells, particularly their regulatory and effector subsets, are crucial for both promoting and regulating immune responses in glioma. Functional modulation of these cells represents an important avenue for therapeutic intervention aimed at reactivating antitumor immunity.

## CD8+ T cell

CD8+ T cells are essential effectors of the immune response against gliomas. Their infiltration into glioma tissues indicates local, antigen-dependent immune activation directed against tumor cells. This infiltration has been linked to antitumor immune responses in gliomas, highlighting their role in the TME [100]. CD8+ T cells also participate in immunoediting during gliomagenesis, influencing genomic stability of glioma cells and shaping the surrounding microenvironment, which can contribute to immune evasion and tumor progression [101]. The abundance and functional state of CD8+ T cells vary significantly within gliomas, with different subtypes exhibiting distinct immune profiles and impacts on tumor biology [102–104]. Notably, CD8+ T-cell presence in the TME is associated with improved survival outcomes, particularly in GBM patients [105]. Higher levels of tumor-infiltrating CD8+ T cells correlate with reduced tumor burden and improved patient prognosis [106]. Immune checkpoint inhibitors (ICIs) such as pembrolizumab have shown promise in treating recurrent high-grade gliomas by enhancing CD8+ T-cell-mediated immune responses. Pembrolizumab treatment has been associated with increased CD8+ T-cell infiltration, particularly in lower-grade gliomas, suggesting that modulation of the immune environment can improve therapeutic efficacy [107]. Furthermore, the roles of various signaling pathways in shaping the glioma immune microenvironment have been explored. The RTK/Ras/PI3K/AKT pathway, for example, has been identified as a potential biomarker for predicting responses to immunotherapy in diffuse glioma, with CD8+ T-cell infiltration serving as a key indicator of immune phenotype and therapeutic response [108]. CD8+ T cells are critical components of the immune landscape in gliomas, and their infiltration constitutes a positive prognostic factor. Enhancing CD8+ T-cell function through immunotherapy holds significant promise for improving glioma treatment outcomes.

## Natural killer (NK) cells

NK cells are essential effectors of innate immunity and show promise in the treatment of GBM. Despite suppression by the TME, NK cells retain the ability to infiltrate glioma tissues, making them a critical target for immunotherapy [109]. Although NK cells frequently infiltrate gliomas, they often undergo functional silencing within the tumor, contributing to the immunosuppressive environment [110]. Research has identified an NK-cell–associated gene signature that serves as a prognostic marker for glioma malignancy and patient survival, further emphasizing NK-cell roles in glioma progression [111]. NK cells mediate immune surveillance and inhibit tumor growth by directly targeting tumor cells [112]. Additionally, immune checkpoint markers such as CD161, expressed on NK cells, have been linked to glioma prognosis, indicating that NK-cell activity can be modulated by specific molecular interactions with the tumor [113]. Adoptive NK-cell therapy has shown encouraging results in preclinical and clinical trials [114]. Ex vivo-expanded NK cells combined with conventional treatments such as temozolomide have demonstrated significant antitumor effects in GBM [115]. However, certain molecular factors, such as GALNT7, impair NK-cell cytotoxicity, leading to sustained glioma cell proliferation and poorer outcomes [116]. This highlights the need for strategies to enhance NK-cell function or overcome these inhibitory mechanisms in glioma treatment.

## B cells

B cells also play a significant role in shaping the glioma TME. Studies demonstrate that B cells are enriched in the glioma-initiating cell niche, suggesting their involvement in glioma progression [117]. Recent evidence highlights the role of regulatory B cells (Bregs) in gliomas, identifying specific B-cell subpopulations that contribute to the immunosuppressive landscape within the tumor [118]. These Bregs can be influenced by factors secreted by glioma cells, such as placental growth factor, which can convert tumor-infiltrating B cells into Bregs, thereby suppressing CD8+ T-cell responses [119]. This B cell-mediated immune suppression is an important aspect of the TME and represents a potential target for therapeutic strategies aimed at reactivating the antitumor immune response.

## Endothelial cells

Endothelial cells play a crucial role in promoting angiogenesis and in providing niches that support glioma growth and progression. Glioma perivascular niches, composed of endothelial cells, are crucial for maintaining glioma stem-cell self-renewal and contribute to the tumor’s chemoresistance [120]. These endothelial cells interact with tumor cells through various signaling pathways that promote tumor progression. Molecular factors such as miR-26a, HCG11, and PVT1 regulate endothelial cell behavior in the glioma microenvironment. For instance, miR-26a is often amplified in glioma tissues and promotes angiogenesis by stimulating microvessel endothelial cell growth [121]. HCG11 acts as a competing endogenous RNA by sponging miR-496, thereby upregulating cytoplasmic polyadenylation element binding protein 3 (CPEB3) in glioma cells [122]. These interactions drive tumor progression by activating downstream signaling pathways that promote endothelial cell proliferation, angiogenesis, and glioma cell growth [123].

## Cell-to-cell communications

Cell-to-cell communication in gliomas plays a crucial role in the progression and aggressiveness of brain tumors. When migrating collectively, glioma cells retain adhesive junctions and intercellular signaling, resembling stable glioma cell networks [124]. This underscores the importance of cell adhesion and intercellular signaling in facilitating communication among glioma cells. Moreover, glioma cells actively interact with surrounding healthy cells and the immune environment, promoting oncogenic processes and contributing to glioma stem cell formation, thereby driving therapy resistance [125].

## Clinically established immunotherapies

Immunotherapy has emerged as a promising treatment strategy for gliomas, particularly given the tumor’s notorious resistance to conventional treatments such as surgery, chemotherapy, and radiotherapy [126]. Several approaches are under investigation, including immune checkpoint inhibitors (ICIs), chimeric antigen receptor T-cell (CAR-T) therapies, vaccines, oncolytic viruses (OVs), monoclonal antibody-mediated immunotherapy, cytokine therapy, adoptive cell transfer, and nanotechnology-mediated immunotherapy [127]. An overview of ongoing and completed clinical trials illustrating these diverse strategies is provided in Table 2. These therapies aim to activate the patient’s immune system to selectively target and eliminate glioma cells [128]. The clinical efficacy of representative immunotherapy trials, including overall survival and progression-free survival outcomes across different strategies, is summarized in Table 3. However, the highly immunosuppressive nature of the glioma microenvironment remains a significant hurdle to successful immunotherapy [129].

**Table 2:** Ongoing and completed clinical trials of immunotherapies in gliomas

Drug and Target	Trial	Phase	Allocation	Treatment	Type of disease	Number of subjects
Pembrolizumab PD-1	NCT02287428	1	Randomized	Radiation Therapy Personalized NeoAntigen Peptides Pembrolizumab Temozolomide Poly-ICLC	Glioblastoma	56
Nivolumab PD-1	NCT03718767	2	N/A	Nivolumab	Glioma	70
MBG453 TIM-3	NCT03961971	1	N/A	MBG453	Glioblastoma	16
Pembrolizumab PD-1	NCT04201873	1	Randomized	Dendritic Cell Tumor Cell Lysate Vaccine Pembrolizumab Poly ICLC	Recurrent Glioblastoma	40
Tecentriq PD-L1	NCT04808245	1	N/A	Tecentriq 1200 MG in 20 ML Injection H3K27M peptide vaccine Imiquimod (5%)	Newly Diagnosed H3-mutated Glioma	15
Anti-CTLA-4 CTLA-4	NCT04943848	1	Non-Randomized	rHSC-DIPGVax Balstilimab Zalifrelimab	Diffuse Intrinsic Pontine Glioma Diffuse Midline Glioma, H3 K27M-Mutant	36
Pembrolizumab PD-1	NCT04977375	1 and 2	N/A	Pembrolizumab Stereotactic Radiation: Surgical Resection	Glioblastoma Multiforme	10
Atezolizumab PD-L1	NCT05039281	1 and 2	N/A	Atezolizumab Cabozantinib	Recurrent Glioblastoma	6
Retifanlimab PD-1	NCT05083754	1	Randomized	A: Retifanlimab and Radiation Therapy B: Retifanlimab, Radiation Therapy and Temozolomide C: Radiation Therapy and Temozolomide	Glioblastoma Multiforme	50
C5252 PD-1	NCT05095441	1	Non-Randomized	C5252	Recurrent or Progressive Glioblastoma	51
Pembrolizumab PD-1	NCT05235737	4	Randomized	Pembrolizumab	Newly Diagnosed Glioblastoma	36
Atezolizumab PD-L1	NCT05423210	1	N/A	Atezolizumab plus FSRT radiation	Glioblastoma Multiforme	12
Tislelizumab PD-1	NCT05502991	2	Non-Randomized	Tislelizumab plus Bevacizumab	Glioblastoma	60
Tislelizumab PD-1	NCT05540275	2	Non-Randomized	Tislelizumab plus Bevacizumab	Recurrent Glioblastoma	30
Tris-CAR-T cells PD-1	NCT05577091	1	N/A	Autologous Tris-CAR-T cells	Glioblastoma	10
Retifanlimab PD-1	NCT05743595	1	Non-Randomized	Personalized Neoantigen DNA vaccine Retifanlimab TDS-IM v 2.0 electroporation device	Unmethylated Glioblastoma	12
Tislelizumab PD-1	NCT05811793	N/A	N/A	Tislelizumab plus Bevacizumab	Glioblastoma	36
PD-L1 t-haNK PD-L1	NCT06061809	2	N/A	Bevacizumab PD-L1 t-haNK *N*-803	Glioblastoma	20
Sintilimab PD-1	NCT06220552	2	N/A	Sintilimab Low-dose Radiotherapy	Recurrent Glioblastoma	20
Pembrolizumab PD-1	NCT06157541	1 and 2	Non-Randomized	Allogeneic cytomegalovirus-specific T cells Pembrolizumab	Glioblastoma Multiforme Astrocytoma Grade IV	58
Pembrolizumab PD-1	NCT06640582	1 and 2	N/A	Tumor Infiltrating Lymphocytes (TILs) Cyclophosphamide Fludarabine Interleukin-2 Pembrolizumab	Glioma	85
Pembrolizumab PD-1	NCT06749925	3	Randomized	Dendritic Cell Vaccine Pembrolizumab Placebo	Glioblastoma	186
BC008-1A PD-1 & TIGIT	NCT06773481	1	Randomized	BC008-1A	Glioma	40
Nivolumab PD-1 Relatlimab LAG-3	NCT06816927	2	Randomized	A:Radiotherapy,Temozolomide B:Nivolumab,Radiotherapy,Temozolomide C:Nivolumab,Relatlimab,Radiotherapy,Temozolomide	Glioblastoma	56
Ipilimumab CTLA-4 Pembrolizumab PD-1	NCT06047379	1 and 2	Non-Randomized	NEO212 Oral Capsule Ipilimumab Pembrolizumab Nivolumab Regorafenib Carboplatin Paclitaxel FOLFIRI Protocol Bevacizumab	Glioblastoma	134
Ipilimumab CTLA-4 Nivolumab PD-1	NCT04003649	1	Randomized	IL13Ralpha2-specific Hinge-optimized 4-1BB-co-stimulatory CAR/Truncated CD19-expressing Autologous TN/MEM Cells Ipilimumab Nivolumab	Recurrent Glioblastoma Refractory Glioblastoma	60
Ipilimumab CTLA-4 Nivolumab PD-1	NCT04396860	2 and 3	Randomized	A:Radiotherapy,Temozolomide B:Radiotherapy,Ipilimumab,Nivolumab	Newly diagnosed MGMT-unmethylated glioblastoma	159
Nivolumab PD-1 Ipilimumab CTLA-4	NCT04145115	2	Randomized	A: Nivolumab B: Nivolumab,Ipilimumab	High mutational burden recurrent glioblastoma	70
Nivolumab PD-1 Ipilimumab CTLA-4	NCT02017717	2	Randomized	A: Nivolumab B: Bevacizumab	Recurrent glioblastoma	369
Nivolumab PD-1 Ipilimumab CTLA-4	NCT03367715	2	Non-Randomized	Nivolumab,Ipilimumab,Short-course Radiotherapy	Newly diagnosed MGMT-unmethylated GBM	30
Nivolumab PD-1 Ipilimumab CTLA-4	NCT06097975	1	Non-Randomized	A:Intravenous Nivolumab,Intracranial Ipilimumab B:Intravenous,Intracranial Nivolumab,Ipilimumab	Recurrent glioblastoma	18
Nivolumab PD-1 Ipilimumab CTLA-4	NCT04817254	2	Non-Randomized	A: Ipilimumab,Nivolumab,Temozolomide B: Temozolomide	Newly diagnosed glioblastoma/gliosarcoma	40

N/A *-Not Applicable

**Table 3:** Efficacy outcomes of selected immunotherapy clinical trials in gliomas

Drug & Target	Trial	Phase	Allocation	Treatment	Type of disease	Overall Survival	Progression- Free Survival	Number of subjects
Nivolumab PD-1	NCT02529072	1	Randomized	Nivolumab DC	Malignant Glioma Astrocytoma Glioblastoma	8.0 vs 15.4	4.3 vs 6.3	6
Nivolumab PD-1	NCT02960230	1 and 2	Non-Randomized	K27M peptide Nivolumab	Diffuse Intrinsic Pontine Glioma Glioma Diffuse Midline Glioma, H3 K27M-Mutant	42	N/A	50
Nivolumab PD-1	NCT03925246	2	N/A	Nivolumab	High Grade Glioma Brain Cancer	N/A	N/A	43
Nivolumab PD-1	NCT03493932	1	N/A	Nivolumab BMS- 986,016	Glioblastoma	N/A	N/A	21
Nivolumab PD-1	NCT02526017	1	Non-Randomized	Cabiralizumab Nivolumab	Glioma	N/A	N/A	313
Varlilumab and Nivolumab PD-1	NCT02335918	1 and 2	N/A	Combination of Varlilumab and Nivolumab	Glioblastoma	N/A	N/A	175
Anti-PD-1 PD-1	NCT02658981	1	Non-Randomized	Anti-LAG-3 Monoclonal Antibody BMS 986,016| Anti-PD-1 Anti-CD137	Glioblastoma	N/A	N/A	63
Pembrolizumab PD-1	NCT03726515	1	N/A	CART-EGFRvIII T cells Pembrolizumab	Glioblastoma	N/A	N/A	7
Pembrolizumab PD-1	NCT04013672	2	Non-Randomized	Pembrolizumab SurVaxM Sargramostim Montanide ISA 51	Recurrent Glioblastoma	N/A	34.5	41
Pembrolizumab PD-1	NCT02798406	2	N/A	DNX-2401 Pembrolizumab	Glioma	N/A	N/A	49
Pembrolizumab PD-1	NCT03797326	2	Randomized	Pembrolizumab Lenvatinib	Glioblastoma	N/A	N/A	603
Bevacizumab, lomustine PD-1	NCT01860638	2	Randomized	Bevacizumab Lomustine Placebo Radiotherapy Temozolomide SOC Agent	Glioblastoma	N/A	N/A	296
Avelumab PD-L1	NCT02968940	2	N/A	Avelumab Hypofractionated radiation therapy (HFRT)	Glioblastoma	10.1	4.2	6
Avelumab PD-L1	NCT03291314	2	Non-Randomized	Axitinib Avelumab	Recurrent Glioblastoma	N/A	N/A	52
Avelumab PD-L1	NCT03893903	1	Randomized	IDH1R132H peptide vaccine Avelumab	Malignant Glioma	N/A	N/A	69
Avelumab PD-L1	NCT03341806	1	N/A	Avelumab MRI-guided LITT therapy	Glioblastoma	N/A	N/A	13
Durvalumab PD-L1	NCT02866747	1 and 2	Randomized	Hypofractionated stereotactic radiation therapy Durvalumab	Glioblastoma	N/A	N/A	108
Durvalumab PD-L1	NCT02336165	2	Non-Randomized	Durvalumab Standard radiotherapy Bevacizumab	Glioblastoma	60.0	19.4 vs 15.2 vs 17.2	159
Tremelimumab CTLA-4 Durvalumab PD-L1	NCT02794883	2	Randomized	Durvalumab Laboratory Biomarker Analysis Surgical Procedure Tremelimumab	Malignant Glioma Recurrent Glioblastoma	7.246 vs 11.71 vs 7.703	N/A	36
Ipilimumab CTLA-4	NCT02311920	1	Randomized	Ipilimumab Laboratory Biomarker Analysis Nivolumab Temozolomide	Supratentorial Glioblastoma	N/A	N/A	32
Anti-LAG-3 Monoclonal Antibody BMS 986,016 LAG-3	NCT02658981	1	Non-Randomized	Anti-LAG-3 Monoclonal Antibody BMS 986016 Anti-PD-1 Pharmacological Study Laboratory Biomarker Analysis Anti-CD137	Glioblastoma Gliosarcoma Recurrent Brain Neoplasm	N/A	N/A	63

N/A – Not applicable

## Immune checkpoint inhibitors (ICIs)

ICIs show potential in glioma treatment by blocking immune checkpoints that suppress T-cell activity. Several studies have developed gene signatures and prognostic models to predict ICI efficacy in glioma patients; some models based on tumor mutational burden provide insight into which patients may benefit most [130]. Additionally, NAD+ metabolism-related gene signatures have been proposed to predict responses to ICIs [131]. While ICIs have improved patient survival in various cancers, their impact in gliomas, especially GBM, has been more variable, and preclinical results do not always translate into clinical success [132]. Challenges such as immune evasion and resistance to immunotherapy, often driven by immunosuppressive factors in the glioma microenvironment, complicate ICI outcomes [133, 134].

## Resistance mechanisms and combination strategies in ICIs

Despite encouraging preclinical findings, clinical trials of ICIs in gliomas have largely been disappointing. Multiple resistance mechanisms have been identified, including the immune-cold nature of gliomas with poor baseline T-cell infiltration; an immunosuppressive TME dominated by TAMs and Tregs, the restrictive BBB; and the generally low tumor mutational burden of gliomas, which limits neoantigen availability [135]. Furthermore, glioma cells can adaptively upregulate alternative checkpoints such as TIM-3 and LAG-3, further suppressing antitumor immunity [136].

To overcome these barriers, combination strategies are actively under investigation. Radiotherapy and OVs may enhance antigen release and T-cell infiltration; TAM reprogramming with CSF1R inhibitors or CAR-M approaches seeks to remodel the immunosuppressive niche; dual checkpoint blockade (e.g., PD-1 with CTLA-4, TIM-3, or LAG-3) may achieve synergistic effects; and the integration of ICB with dendritic cell or peptide vaccines could further amplify antigen presentation [137]. In addition, novel nanomedicine platforms and BBB-modulating strategies are under development to facilitate more effective immune- and drug-penetration into the CNS [138]. Collectively, these approaches may help unlock the full therapeutic potential of ICB in gliomas when applied in rational, multimodal combinations.

## Chimeric antigen receptor T cell (CAR-T) therapy

CAR-T therapy has demonstrated significant promise in treating hematologic cancers and is being explored for solid tumors, including GBM. Despite successes in preclinical models and clinical trials, tumor relapse remains common owing to various resistance mechanisms [139]. Several studies are investigating CAR-T therapies targeting specific glioma antigens. For example, GD2, which is highly expressed in H3K27M-mutated gliomas, has been the focus of a phase I trial using GD2-directed CAR-T cells [140]. However, CAR-T therapy in adult high-grade gliomas faces several challenges, including the immunosuppressive glioma microenvironment and the lack of suitable tumor-specific antigens [141]. Strategies such as IL-15 expression have shown promise in enhancing CAR-T efficacy in GBM, though antigen heterogeneity remains a significant obstacle [142]. Additionally, challenges such as on-target off-tumor toxicity and potential immunodeficiency risks require careful consideration [143, 144].

## Chimeric antigen receptor macrophage (CAR-M) therapy

CAR-M therapy is an emerging approach designed to address limitations of CAR-T cell therapies. By engineering macrophages with chimeric antigen receptors, CAR-M therapy aims to enhance phagocytic activity and directly target tumor-associated antigens [145]. This strategy leverages both innate and adaptive immunity, rendering it a promising option for treating gliomas [146]. CAR-M therapy has shown promising preclinical results, demonstrating the capacity to overcome challenges such as tumor infiltration and the immunosuppressive microenvironment [147]. Innovations such as cavity-injectable nanoporter-hydrogel systems have been used to develop GSC-specific CAR macrophages, which could help prevent GBM recurrence [148].

## Bispecific antibodies (BsAbs)

BsAbs are designed to bind two distinct antigens simultaneously, redirecting immune cells toward glioma cells. The dual-targeting strategy has shown promise in reprogramming TAMs and delaying tumor growth in glioma models. For example, BsAbs targeting angiopoietin-2 (Ang-2) and vascular endothelial growth factor (VEGF) have altered TAM activity in preclinical glioma models [149]. BsAbs such as anti-CD3×anti-HER2/neu and/or anti-CD3×anti-EGFR have shown effectiveness in targeting GBM tumor cells expressing HER2/neu and EGFR, respectively [150]. The design of fully human BsAbs, such as hEGFRvIII-CD3 bi-scFv, has demonstrated the ability to redirect T cells to target gliomas with specific mutations, offering a promising therapeutic approach for GBM [151]. BsAbs have been used to enhance gene transfer and adenoviral gene delivery, potentially improving the efficacy of targeted therapies [152].

## Further perspective

Advances in understanding the glioma TME underscore the potential of immunotherapy as a promising therapeutic avenue. High-grade gliomas, particularly GBM, are characterized by a profoundly immunosuppressive TME that limits the effectiveness of conventional approaches such as surgery, radiotherapy, and chemotherapy. While progress with ICIs and CAR-T therapies has demonstrated that gliomas can be immunologically targeted, durable clinical benefit remains limited. Advancing the field requires not only evaluation of current barriers but also articulation of potential therapeutic directions, addressing persistent challenges, and incorporation of novel approaches on the horizon.

One emerging direction is the development of adoptive cell therapies beyond conventional CAR-T. Although CAR-T cells have shown activity in hematologic malignancies, their efficacy in gliomas is hindered by antigen heterogeneity, restricted trafficking, and local immunosuppression. Expanding adoptive platforms to include NK cells, γδ T cells, and tumor-infiltrating lymphocytes (TILs) may help overcome these obstacles. NK cells possess innate cytotoxic activity that does not depend on prior antigen sensitization, making them attractive for treating heterogeneous glioma populations. Similarly, TILs enriched from glioma tissue could exploit patient-specific immune repertoires. Major challenges include manufacturing processes, ensuring persistence within the CNS, and managing safety profiles.

Another promising avenue is the use of OVs, which selectively infect and lyse tumor cells while simultaneously stimulating antitumor immunity. In gliomas, OVs provide dual benefits: direct cytotoxicity and immune priming. Viral platforms such as adenovirus, herpes simplex virus, and poliovirus derivatives have advanced to clinical testing with encouraging safety signals and preliminary efficacy. However, challenges remain, including efficient delivery across the BBB, potential neutralization by host immunity, and the risk of CNS inflammation. Combining OVs with ICIs or adoptive cell therapies may enhance immunogenicity and mitigate resistance mechanisms.

A third frontier involves personalized neoantigen vaccines. By leveraging patient-specific tumor mutations, these vaccines aim to elicit robust T-cell responses against unique neoepitopes. In gliomas, where shared antigens are limited and heterogeneity is high, neoantigen-based vaccines offer a highly individualized therapeutic option. Advances in next-generation sequencing, epitope prediction, and vaccine platforms, including peptide, RNA, and dendritic cell–based vaccines, are accelerating clinical translation. Challenges remain in sustaining immune activity within the suppressive TME, managing production timelines, and selecting optimal combination strategies with ICIs or cytokine support.

Despite these innovations, significant challenges persist. Gliomas are shaped by diverse immune cell subsets, tumor antigen heterogeneity, and potent immunosuppressive factors such as myeloid-derived suppressor cells, Tregs, and abnormal vasculature. Moreover, the BBB and the risk of neurotoxicity impose unique safety considerations. These factors underscore the need for multipronged strategies rather than reliance on single modalities.

Looking forward, the future of glioma immunotherapy will likely depend on rationally designed combinations that target both the tumor and its microenvironment. For instance, pairing adoptive NK or TIL therapy with OVs could enhance immune infiltration, whereas integrating personalized vaccines with ICIs may sustain antitumor activity. Advances in biomarker discovery will be crucial for guiding patient selection, monitoring therapeutic response, and anticipating resistance. Cutting-edge technologies such as single-cell sequencing, spatial transcriptomics, and artificial intelligence–driven modeling will further refine understanding of the TME and enable precision strategies.

In conclusion, although current immunotherapies for gliomas face substantial obstacles, the field is steadily advancing toward more effective and durable options. Adoptive cell therapies, OVs, and personalized neoantigen vaccines exemplify next-generation approaches that could complement existing modalities. By addressing the immunosuppressive nature of the TME and integrating innovative platforms into multipronged therapeutic frameworks, future research holds promise for improving survival and quality of life for patients with these challenging brain tumors.

## Data Availability

No datasets were generated or analysed during the current study.
